# The impact of laxative use upon symptoms in patients with proven slow transit constipation

**DOI:** 10.1186/1471-230X-11-121

**Published:** 2011-11-10

**Authors:** Phil G Dinning, Linda Hunt, David Z Lubowski, Jamshid S Kalantar, Ian J Cook, Mike P Jones

**Affiliations:** 1 Department of Human Physiology, School of Medicine. Flinders University. Adelaide, SA. 5042. Australia; 2 St. George Clinical School, University of New South Wales, Sydney, NSW. 2217 Australia; 3 Ano-rectal Physiology. Sydney Colorectal Associates, Dept Colorectal Surgery. St. George Hospital. Sydney, NSW. 2217 Australia; 4 The Whiteley-Martin Research Centre, The Discipline of Surgery, The University of Sydney, Sydney Medical School, Nepean Hospital, Penrith, NSW 2751, Australia; 5 Gastroenterology and Hepatology, St.George Hospital, Sydney & University of New South Wales. Sydney. NSW. 2217. Australia; 6 Psychology Dept. Macquarie University. Sydney. NSW. 2019. Australia

## Abstract

**Background:**

Constipation severity is often defined by symptoms including feelings of complete evacuation, straining, stool frequency and consistency. These descriptors are mostly obtained in the absence of laxative use. For many constipated patients laxative usage is ubiquitous and long standing. Our aim was to determine the impact of laxative use upon the stereotypic constipation descriptors.

**Methods:**

Patients with confirmed slow transit constipation completed 3-week stool diaries, detailing stool frequency and form, straining, laxative use and pain and bloating scores. Each diary day was classified as being under laxative affect (laxative affected days) or not (laxative unaffected days). Unconditional logistic regression was used to assess the affects of laxatives on constipation symptoms.

**Results:**

Ninety four patients with scintigraphically confirmed slow transit constipation were enrolled in the study. These patients reported a stool frequency of 5.6 ± 4.3 bowel motions/week, only 21 patients reported <3 bowel motions/week. Similarly, 21 patients reported a predominant hard stool at defecation. The majority (90%) of patients reported regular straining. A regular feeling of complete evacuation was reported in just 7 patients. Daily pain and/or bloating were reported by 92% of patients. When compared with laxative unaffected days, on the laxative affected days patients had a higher stool frequency (OR 2.23; *P *<0.001) and were more likely to report loose stools (OR 1.64; *P *<0.009). Laxatives did not increase the number of bowel actions associated with a feeling of complete evacuation. Laxative use had no affect upon straining, pain or bloating scores

**Conclusions:**

The reporting of frequent and loose stools with abdominal pain and/or bloating is common in patients with slow transit constipation. While laxative use is a significant contributor to altering stool frequency and form, laxatives have no apparent affect on pain or bloating or upon a patients feeling of complete evacuation. These factors need to be taken into account when using constipation symptoms to define this population.

## Background

Severe constipation is a chronic condition with major morbidity and health care burden [1,2]. As symptoms of constipation are the first points of discussion between a patient and their clinician the ability to subtype patients based upon these symptoms is important because, ideally it should direct a logical and cost-effective investigative pathway without the need for expensive and inconvenient testing.

Two of the most commonly identified symptoms of constipation are infrequent and hard lumpy stools. Both of these characteristics are fundamental to major current consensus-derived definitions of constipation such as the Rome III criteria [3] and the American College of Gastroenterology Chronic Constipation Task Force [4]. The Rome III criteria also include straining, a feeling of incomplete evacuation and manual maneuvers to facilitate defecation. It is noteworthy that all these criteria are established in the absence of laxative use. Indeed the Rome criteria qualified by the statement that loose stools are rarely present without the use of laxatives [3].

However, the ability to critically and realistically assess constipation symptoms in the absence of laxative use can be difficult in a chronically constipated population. Laxatives remain a primary treatment option in such patients [5,6] and in many instances patients may have been taking laxatives for many years. Therefore when asked at interview or in a questionnaire to detail their stooling characteristics in the absence of laxatives they have to do so from memory. Responses based purely on recall of stooling habits have been shown to be unreliable [7-9].

The use of stool diaries avoids the problems associated with patients' recall ability [9-16]. However, in systematic studies of constipation these diaries are usually kept with patients off laxatives [8,13]. As laxative usage can potentially alter stool frequency and consistency, the stereotypic classifier of infrequent hard stools in severe constipation may bear little or no resemblance to the reality of the day-to-day life in these patients.

This question is important to the clinician and the researcher, particularly when trying to quantify stooling habits and outcomes in clinical trials. There is a need to try and classify constipated patients according to subtype [17,18]. This is best achieved by physiological function tests of colonic transit and anorectal evacuation. These tests are costly, inconvenient and not always readily available. Currently, based on existing symptom measurement tools, prediction of the physiological measures is imperfect [5]. It is unknown whether laxative-related perturbation of symptoms may contribute to this lack of predictive value of symptoms for measureable mechanical dysfunction.

There has never been a systematic evaluation of the potential impact of laxative usage on symptoms in patients with constipation. Utilising a 3-week stool diary completed by patients with slow transit constipation, our aim was to evaluate, on a day-to-day basis, this relationship and determine the specific impact of laxative use, if any, upon stool frequency and form, straining, feelings of complete evacuation in addition to reported instances of abdominal pain and bloating. Specifically we hypothesised that all of these symptoms are affected by laxative use in patients with slow transit constipation.

## Methods

### Population sample

Patients included in this study were referred to tertiary centres for potential inclusion in a trial of the treatment of constipation with sacral nerve stimulation. For the initial inclusion in the trial patients had to meet the following inclusion criteria; i) aged 18 - 75 yrs; ii) deemed have Rome III criteria for constipation [3] (assessed by DZL and IJC); iii) a failed response to standard therapies including laxatives, dietary modification and exercise; and iv) Confirmed slow transit constipation [19]. Patients were excluded from the study if they had any of the following; i) metabolic, neurogenic or endocrine disorder(s) known to cause constipation (e.g. hypercalcaemia, hypothyroidism, diabetes, multiple sclerosis, Parkinson's, scleroderma); ii) consumed drugs which list constipation as a potential side effect deemed to be clinically relevant by the referring physician (e.g. calcium channel blockers); iii) prior abdominal radiotherapy; iv) prior abdominal surgery (except cholecystectomy, appendicectomy, inguinal hernia repair); v) current pregnancy; vi) current or prior history of malignancy.

All participants meeting these criteria gave written, informed consent and the studies were approved of in Australia by the Human Ethics Committees of the South Eastern Area Health Service, Sydney and the University of New South Wales (05/122)

### Stool diary

Once patients met the inclusion criteria they were given stool diaries to complete. The stool diary is based on an instrument used for many years in our laboratory. For the present study questions of specific interest have been added concerning "feeling of incomplete evacuation" as well as the laxative use, type and dose. Each stool diary consisted of 3 pages, 1 for each week of the study. On each day patients were requested to enter; i) their stool frequency (0,1,2,≥3); ii) the stool form of each bowel action (based upon the Bristol stool scale [20], ranging from 1 = separate hard lumps, like nuts; 4 = like a sausage or snake smooth and soft; 7 = watery, no solid pieces); iii) straining (yes/no); iv) a sense of complete evacuation (yes/no); v) pain and bloating scores (0 = none, 1 = present but tolerable, 2 = present and interfering with but not preventing normal daily activities like work and sleep, 3 = preventing normal daily activities); vi) laxative use (yes/no) and if yes what type and dose. In a cover sheet patients were also asked how long they had had constipation. Completed diaries were placed into a self addressed prepaid envelope and returned by post to the investigators.

### Colonic transit studies

Colonic transit was measured using a standard nuclear medicine technique previously validated at St. George Hospital [21,22]. All laxatives were stopped 3 days before the scan and during scanning period. The protocol and analysis of the colonic scintigraphy have been described previously [17,23]. The scintigraphic definition of delayed transit constipation was met if the study showed isotope retention of greater than 9% in the right (caecum to mid transverse) or left colon (mid-transverse to distal descending colon) at 72 hrs [21,22].

### Data analysis & statistics

Since the effect of laxative use is likely to be relatively short-lived we decided against averaging data over the three-week period. Instead we looked at the data at a daily level with each day classified as a day on which laxatives were in affect or not. The term "laxative-affect days" incorporates the day on which the laxatives were taken as well as the following day since a laxative taken late on one day was likely to have some carry over effect into the following day. We have used the term "laxative unaffected days" to define all other days. The affect of laxative use on bowel habit was then assessed via unconditional logistic regression with standard errors adjusted using the linearization method [24] to account for within-subject correlation which involves adjusting the naive standard errors by a variance inflation factor. Odds ratios from the model are used to interpret the direction and effect size of laxatives on bowel habit and are reported with 95% confidence intervals and p-values. An odds ratio >1 indicates that laxative effect is associated with higher levels of a bowel habit variable while odds ratios <1 indicate an association with lower levels of that variable. An odds ratio of exactly 1.0, or very close to it, indicates no effect of laxatives on that bowel habit variable.

#### Stool Form

As reported previously [13] the Bristol stool form scale was reduced to a three-point scale by combining scores of 1 and 2 into a "hard" group (Type 1), scores 3 and 4 as a "normal" group (Type 2) and scores of 5-7 as a "soft" group (Type 3).

#### Laxative Use

The patients in this study used a wide variety and combination of laxatives. We defined a laxative as an agent specifically designed to stimulate the evacuation of faeces. The vast majority of laxatives used fell into two specific groups; i) Stimulant laxatives (Senna, Bisacodyl, Coloxyl, Durolax, Aloe); ii) Osmotics Laxatives (PEG, Milk of Magnesia, Magnesium sulfate). Twenty of the patients also used enemas. In 18 patients enemas were used on ≤5 occasions during the 3-week period and always in combination with other laxatives. In 2 patients enemas were used without any other laxatives. These two patients used the enemas on every 2nd or 3rd day respectively. The laxatives were also occasionally supplemented with bulking agents such as prunes or bran. The wide variety of laxative doses and combinations prevented any meaningful statistical comparison between specific laxative type/dose and symptoms.

## Results

**Figure 1:**
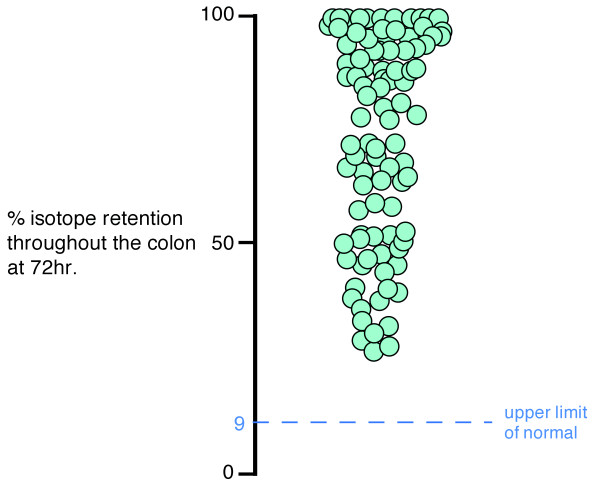
**The percent retention of isotope in the colon at 72 hr**.

A total of 94 patients (5 male; mean age 43 ± 17 yrs) with scintigraphically confirmed slow transit were enrolled in the study. As these were patients being selected for possible inclusion in a trial for treatment of constipation with sacral nerve stimulation the response rate was excellent, with all patients completing the diaries. The average colonic retention at 72 h was 73.9%. ± 25% (Figure 1). The majority of patients (n = 85; 91%) reported having suffered from constipation for longer than 5 years (62% for longer than 10 yrs). The remaining 9 patients reported constipation for at least 2 years.

### Stool Diary

**Figure 2:**
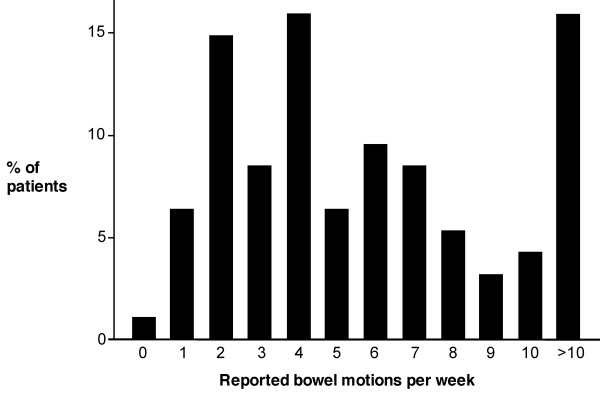
**Weekly stool frequency reported by the patients with slow transit constipation**.

Laxative use was prominent in the majority of patients with 28 (30%) using laxatives on a daily basis, 49 (52%) using laxatives intermittently and 17 (18%) reporting no laxative use. The stool frequency collated from the stool dairies was 5.6 ± 4.3 bowel motions per week. Only 21 (22%) patients reported a stool frequency of <3 bowel motions/week with 35 (37%) reporting at least one bowel motion per day (Figure 2). A predominant hard stool at defecation was reported by 21 (22%) patients, while 28 (30%) reported loose stool. Straining during a bowel action was common with 30 (32%) having to strain for every bowel action and only 10 (11%) reporting that they never strain. Despite the frequency of the reported bowel motions 56 (60%) patients never (or rarely) reported a feeling of complete evacuation. Indeed a regular feeling of complete evacuation was reported in just 7 (8%) patients. Daily pain or bloating scored as interfering or preventing normal daily activities was reported by 27 (28.7%) and 40 (42.5%) patients, respectively. Less than 8% of patients reported no pain or bloating.

### Laxative affected and unaffected days

**Figure 3:**
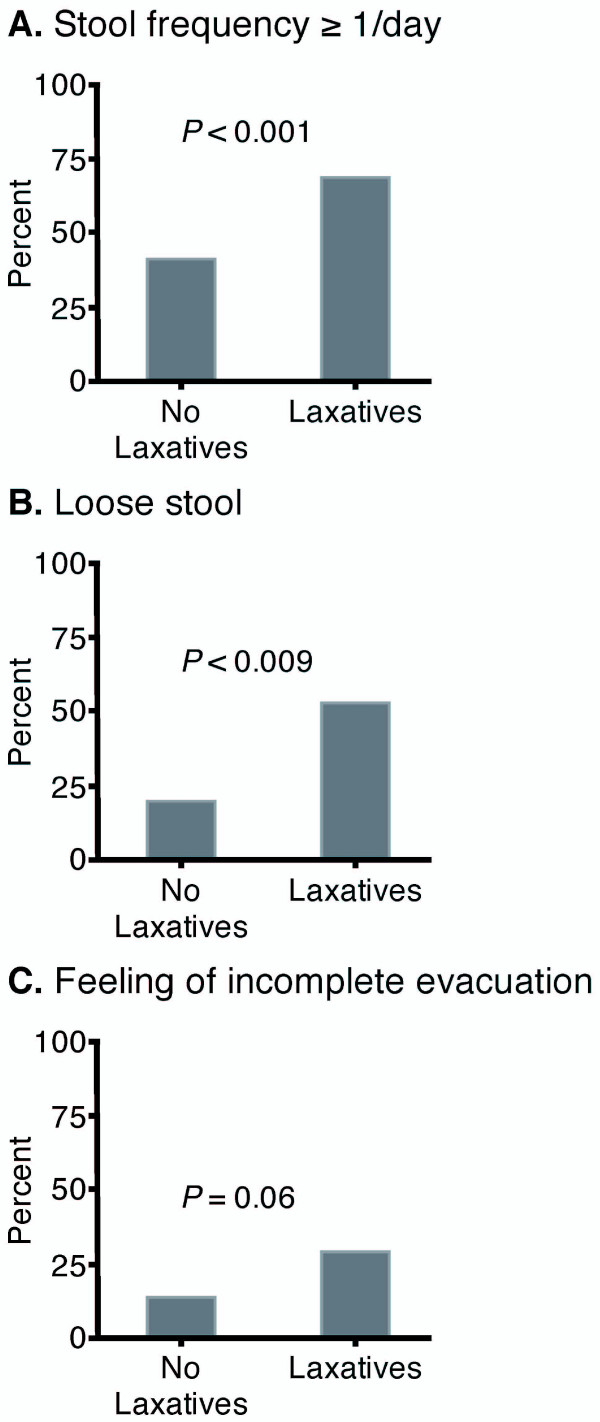
**The percentage of days on which; A) at least one bowel action was passed; B) a loose stool was passed; and C) a feeling of complete evacuation was achieved, during the laxative affected and unaffected days**. Stool frequency and incidence of loose stool were significantly increase on laxative affected days.

These patients yielded 1051 diary days; 497 laxative-affect days and 554 laxative unaffected days. The statistical comparisons between stooling characteristics on laxative affected and unaffected days are shown in table 1 and Figure 3. A stool frequency of ≥1 bowel motion per day occurred on 70% of the laxative affected days and 41% of the laxative unaffected days (*P *< 0.0001). Similarly on 54% of laxative affected days patients reported loose stool compared to the 28% of laxative unaffected days (*P *< 0.0001). A feeling of complete evacuation was slightly increased on laxative affected days, however this difference was not significant (table 1). Laxative use had no effect upon straining, pain or bloating scores (table 1).

**Table 1:** These odds ratios suggest that among patients with demonstrated delayed colonic transit days on which laxatives were either taken or likely to have been in effect are differentiated from laxative unaffected days with respect to stool form (higher scores on laxative days), stool frequency (higher frequency on laxative days) and feeling of complete evacuation (FOCE: more likely on laxative effect days) but are not differentiated with respect to straining, abdominal pain or bloating.

Characteristics	Odds ratio	95% CI	*P - value*
Stool form	1.64	1.13, 2.40	0.009
Stool frequency	2.23	1.57, 3.17	<0.001
FOCE	2.01	0.95, 4.22	0.06
Straining	1.10	0.50, 2.45	0.8
Abdominal pain	1.00	0.77, 1.30	>0.9
Abdominal bloating	1.04	0.78, 1.38	0.8

## Discussion

This study indicates that frequent, loose stools, pain and bloating are all commonly reported in a patient group with confirmed slow colonic transit and constipation. Indeed three quarters of our patients fell within the defined 3/wk - 3/day normal range for stool frequency [25,26] and 78% described normal to loose stool. Straining at stool and a feeling of incomplete evacuation were also commonly reported. Over 80% of our patients regularly took laxatives, and our data indicate that laxative use has a significant impact upon the reported stool frequency and form. However despite increasing the stool frequency laxatives had little affect on a patient's ability to feel as though they had completely evacuated and they did not reduce the incidence of straining. Laxatives also had no apparent affect upon abdominal pain and bloating suggesting that pain and bloating are intrinsic to the constipation.

Laxatives remain the primary treatment option for severe constipation [6] and in the US the annual expenditure on laxatives is estimated to exceed $US850 M [27,28]. Yet despite the common usage their potential impact upon the day-to-day constipation symptoms is often overlooked and this raises potential problems when attempting to critically assess the reality of symptoms in chronically constipation patients. In this study 91% of our patients reported having constipation for at least 5 years (62% >10 yrs) and laxative use for many had become part of every day life. Asking such patients to detail their stooling habits in the absence of laxative use is unlikely to provide realistic data. Indeed in our experience the classic response to the question "how often would you open your bowels a week without laxative use?" is "I'd never go". While such responses may represent the patient's perspective of their bowel function, several studies have shown that a patients recall ability of stool habits is inaccurate [7-9].

These inaccuracies could potentially confound study findings. For example previous studies have suggested that infrequent stools may indicate delayed transit [10,12,29] and some studies have presented supportive evidence for this [30,31], while others have not [9,13]. When looking at the methodology of these studies it is of interest to note that those that associated infrequent stools with delayed transit, based the patient's stool frequency upon answers provided in a questionnaire [30,31]. Thus the acquired data relied on the patients recall ability, presumably in the absence of laxatives, which is more likely to indicate a low weekly stool frequency. Those studies that found no correlation between stool frequency and delayed colonic transit assessed the stool frequency on data obtained in stool diaries [9,13]. While not directly assessed here our data would also suggest that neither stool form nor frequency provides an accurate indicator of slow colonic transit.

To overcome the impact of laxatives upon constipation symptoms, stool diaries could be maintained in the absence of laxatives use, however in our experience chronically constipated patients become dependent on laxatives and can be unwilling to stop using them for even short periods of time. Such patients can be excluded from studies assessing constipation symptoms [8] but then these studies risk removing patients at the more severe end of constipation scale.

The potential for laxatives to alter stool frequency and form can also have implications for studies that use constipation symptoms in an attempt to define constipation subtypes. The mathematical techniques of factor analysis have been used in an attempt to determine whether certain symptoms groups can help to define subtypes of constipation. Studies applying these techniques have found conflicting results with Mertz et al [32] identifying 3 constipation subtypes while Eltringham el at [33] did not. In our own recent study, 4 subtypes were identified, however there was significant overlap amongst these subtypes [17]. Given that the approach to constipation subtyping in our study relied entirely on answers provided in a questionnaire, it is important that patients understand how they are supposed to complete the questionnaire. For example we have had many patients ask if they should complete the questionnaire based on what they are like on or off laxatives. Therefore within any population of constipated patients the questionnaires may be assessing; i) a patient's estimate of what they believe they would be like without laxatives; ii) what they are actually like on laxatives; or iii) a combination of both situations.

While laxatives in this study increased stool frequency, it is important to note that on the majority of laxative affected days patients still had to strain to open their bowels and were left with a feeling of incomplete evacuation. As a consequence the patient still feels constipated. This highlights a potential problem associated with using stool frequency as a measure of constipation severity. To manoeuvrer around this many studies use 'spontaneous bowel motion' or 'complete spontaneous bowel motion' to define bowel habit [4,34]. However, once again in chronically constipated patients that have become dependant upon laxatives, the reality of obtaining several weeks of stool frequency data in the absence of any laxative use could be difficult. In such patients we would suggest that a more accurate measure of constipation severity could be the number of days per week in which a patient achieves a feeling of complete evacuation in light of their laxative use. In our patients, while many fit the 'normal' stool frequency criteria, the vast majority experienced a bowel movement associated with a feeling of complete evacuation on less than 3 days per week.

An important and often overlooked factor to emerge from our stool diaries is the prevalence of pain and bloating. Frequent, loose stools, abdominal pain and bloating are all symptoms commonly associated with irritable bowel syndrome, and the current Rome criteria for the diagnosis of constipation does not even mention the presence of pain or bloating [3]. Only 8% of our patients reported an absence of these symptoms whereas >27% reported these symptoms as interfering or preventing normal daily activities. Previous studies have shown a large proportion of patients with constipation, including those with slow transit constipation, potentially met the Rome criteria for IBS [17,35,36], thus highlighting the need to include pain and bloating in the assessment of constipation symptoms.

There are potential criticisms with our study. Straining and pain/bloating, reported by 89% and 92% of our patients, respectively are commonly reported by patients who have a disorder of rectal evacuation [37,38]. Given that tests of anorectal function were not used to exclude patients with co-existent disordered rectal evacuation, it is likely that such mixed disorders existed in some of our study population and this could potentially impact upon our results. Indeed the inclusion of such patients has recently been put forward as a potential confounder in clinical trials of prokinetics in constipation [39]. However, this assertion still remains to be proven and as there is a large body of evidence that suggests there are no symptoms that are reliably predictive of an evacuation disorder [35,40-43], we feel that the potential inclusion of such patients should not detract from the overall symptomatic findings in our study.

Other potential criticisms of this study include the fact we did not measure the size or the weight of stools that were passed and we therefore we have no ability to determine what a patient considers to be a 'bowel motion'. Passing a small amount of hard or liquid stool may have constituted a bowel motion for some patients. Of course this factor remains true for almost all symptom based constipation studies. Finally while there is a risk of non-compliance with reporting the daily symptoms, especially over a three week period, we found that with these particular patients, who were all seeking help for the constipation symptoms, there was a willingness to comply and we are therefore confident that these data are an accurate representation of the symptoms in this population.

## Conclusion

In conclusion these data demonstrate that in the normal day-to-day life of patients with slow transit constipation laxative use significantly increases stool frequency and the incidence of loose stool without reducing the patients need to strain or increasing their ability to feel as though they have evacuated completely. Furthermore, these patients commonly report abdominal pain and bloating, factors that were not affected by laxative use, and are therefore a symptomatic component of severe constipation. We feel that based upon these data; i) stool frequency and form are poor indicators of constipation severity; ii) abdominal pain and bloating should be assessed as part of the constipation symptoms; and iii) daily stool diaries detailing constipation symptoms should include laxative use. The data derived from such dairies provides a more realistic insight into the day-to-day symptoms of patients with constipation.

## Competing interests

The authors declare that they have no competing interests.

## Authors' contributions

PD & MJ; study concept and design, statistical analysis, draft and critical review of manuscript; LH data collation and entry; JK, DL & IC, patient selection and critical manuscript review. All authors all authors read and approved the final manuscript.

## Pre-publication history

The pre-publication history for this paper can be accessed here:

http://www.biomedcentral.com/1471-230X/11/121/prepub

## References

[B1] Everhart JE Ruhl CE Burden of Digestive Diseases in the United States Part I: Overall and Upper Gastrointestinal Diseases Gastroenterology 2009 136 376 386 10.1053/j.gastro.2008.12.015 19124023

[B2] Liem O Harman J Benninga M Kelleher K Mousa H Di Lorenzo C Health utilization and cost impact of childhood constipation in the United States Journal of Pediatrics 2009 154 2 258 262 10.1016/j.jpeds.2008.07.060 18822430

[B3] Longstreth GF Thompson WG Chey WD Houghton LA Mearin F Spiller RC Functional bowel disorders Gastroenterology 2006 130 5 1480 1491 10.1053/j.gastro.2005.11.061 16678561

[B4] Force ACoGCCT An evidence-based approach to the management of chronic constipation in North America Am J Gastroenterol 2005 100 Suppl 1-4 S1 16008640 10.1111/j.1572-0241.2005.50613_1.x

[B5] Emmanuel AV Tack J Quigley EM Talley NJ Pharmacological management of constipation Neurogastroenterol Motil 2009 21 Suppl 2 41 54 19824937 10.1111/j.1365-2982.2009.01403.x

[B6] Singh S Rao SS Pharmacologic management of chronic constipation Gastroenterol Clin North Am 2010 39 3 509 527 10.1016/j.gtc.2010.08.001 20951915

[B7] Manning AP Wyman JB Heaton KW How trustworthy are bowel histories?Comparisonof recalled and recorded information Br Med Journal 1976 2 213 214 10.1136/bmj.2.6029.213 PMC1687305 974496

[B8] Ashraf W Park F Lof J Quigley EM An examination of the reliability of reported stool frequency in the diagnosis of idiopathic constipation Am J Gastroenterol 1996 91 1 26 32 8561138

[B9] Chaussade S Khyari A Roche H Garret M Gaudric M Couturier D Guerre J Determination of total and segmental colonic transit time in constipated patients - results in 91 patients with a new simplified method Dig Dis Sci 1989 34 8 1168 1172 10.1007/BF01537263 2546720

[B10] Davies GJ Crowder M Reid B Dickerson JW Bowel function measurements of individuals with different eating patterns Gut 1986 27 2 164 169 10.1136/gut.27.2.164 3005140 PMC1433202

[B11] Degen LP Phillips SF How well does stool form reflect colonic transit? Gut 1996 39 1 109 113 10.1136/gut.39.1.109 8881820 PMC1383242

[B12] Lewis SJ Heaton KW Stool form scale as a useful guide to intestinal transit time Scand J Gastroenterol 1997 32 9 920 924 10.3109/00365529709011203 9299672

[B13] Saad RJ Rao SS Koch KL Kuo B Parkman HP McCallum RW Sitrin MD Wilding GE Semler JR Chey WD Do Stool Form and Frequency Correlate With Whole-Gut and Colonic Transit? Results From a Multicenter Study in Constipated Individuals and Healthy Controls Am J Gastroenterol 2010 105 2 403 411 10.1038/ajg.2009.612 19888202

[B14] Wyman JB Heaton KW Manning AP Wicks AC Variability of colonic function in healthy subjects Gut 1978 19 2 146 150 10.1136/gut.19.2.146 631630 PMC1411830

[B15] Oettle GJ Effect of moderate excercise on bowel habit Gut 1991 32 941 944 10.1136/gut.32.8.941 1885077 PMC1378967

[B16] O'Donnell LJ Virjee J Heaton KW Detection of pseudodiarrhoea by simple clinical assessment of intestinal transit rate BMJ 1990 300 6722 439 440 10.1136/bmj.300.6722.439 2107897 PMC1662249

[B17] Dinning PG Jones M Hunt L Fuentealba SE Kalanter K King DW Lubowski DZ Talley NJ Cook IJ Factor analysis identifies subgroups of constipation World J Gastroenterol 2011 17 11 1468 1474 10.3748/wjg.v17.i11.1468 21472106 PMC3070021

[B18] Cook IJ Talley NJ Benninga MA Rao SS Scott SM Chronic constipation: Overview and challenges Neurgastroenterol Mot 2009 21 s2 1 8 10.1111/j.1365-2982.2009.01399.x 19824933

[B19] McLean RG King DW Talley NA Tait AD Freiman J The utilization of colon transit scintigraphy in the diagnostic algorithm for patients with chronic constipation Dig Dis Sci 1999 44 1 41 47 10.1023/A:1026689714120 9952221

[B20] Heaton KW Ghosh S Braddon FE How bad are the symptoms and bowel dysfunction of patients with the irritable bowel syndrome? A prospective, controlled study with emphasis on stool form Gut 1991 32 1 73 79 10.1136/gut.32.1.73 1991641 PMC1379218

[B21] McLean RG Smart RC Lubowski DZ King DW Barbagallo S Talley NA Oral colon transit scintigraphy using Indium-111 DTPA: variability in healthy subjects Int J Colorectal Dis 1992 7 173 176 10.1007/BF00341215 1293236

[B22] McLean RG Smart RC Gaston-Parry D Barbagello S Baker J Lyons NR Bruck CE King DW Lubowski DZ Talley NA Colon transit scintigraphy in health and constipation using oral iodine-131-cellulose J Nucl Med 1990 31 985 989 2348244

[B23] Dinning PG Zarate N Hunt LM Fuentealba SE Mohammed SD Szczesniak MM Lubowski DZ Preston SL Fairclough PD Lunniss PJ Pancolonic spatiotemporal mapping reveals regional deficiencies in, and disorganization of colonic propagating pressure waves in severe constipation Neurgastroenterol Motil 2010 22 e340 e349 10.1111/j.1365-2982.2010.01597.x 20879994

[B24] Kish L Survey Sampling 1975 New Yory: Wiley Pty Ltd

[B25] Connell AM Hilton C Irvine G Lennard-Jones JE Misiewicz JJ Variation of bowel habit in two population samples Br Med J 1965 2 5470 1095 1099 10.1136/bmj.2.5470.1095 5838411 PMC1846921

[B26] Drossman DA Sandler RS McKee DC Lovitz AJ Bowel patterns among subjects not seeking health care. Use of a questionnaire to identify a population with bowel dysfunction Gastroenterology 1982 83 3 529 534 7095360

[B27] Rao SS Constipation: evaluation and treatment of colonic and anorectal motility disorders Gastroenterol Clin North Am 2007 3 687 711 10.1016/j.gtc.2007.07.013 17950444

[B28] Levy S Drug outlets remain leader in laxative sales Drug topics 2002 8

[B29] Gorard DA Gomborone JE Libby GW Farthing MJ Intestinal transit in anxiety and depression Gut 1996 39 4 551 555 10.1136/gut.39.4.551 8944564 PMC1383268

[B30] Ducrotte P Rodomanska B Weber J Guillard JF Lerebours E Hecketsweiler P Galmiche JP Colin R Denis P Colonic transit time of radiopaque markers and rectoanal manometry in patients complaining of constipation Dis Colon Rectum 1986 29 10 630 634 10.1007/BF02560322 3757701

[B31] Glia A Lindberg G Quality of life in patients with different types of functional constipation Scand J Gastroenterol 1997 32 11 1083 1089 10.3109/00365529709002985 9399387

[B32] Mertz H Naliboff B Mayer EA Symptoms and physiology in severe chronic constipation Am J Gastroenterol 1999 94 1 131 138 10.1111/j.1572-0241.1999.00783.x 9934743

[B33] Eltringham MT Khan U Bain IM Wooff DA Mackie A Jefferson E Yiannakou Y Functional defecation disorder as a clinical subgroup of chronic constipation: Analysis of symptoms and physiological parameters Scand J Gastroenterol 2008 43 3 262 269 10.1080/00365520701686210 18266173

[B34] Kamm MA Muller-Lissner S Talley NJ Tack J Boeckxstaens G Minushkin ON Kalinin A Dzieniszewski J Haeck P Fordham F Tegaserod for the treatment of chronic constipation: a randomized, double-blind, placebo-controlled multinational study Am J Gastroenterol 2005 100 2 362 372 10.1111/j.1572-0241.2005.40749.x 15667494

[B35] Grotz RL Pemberton JH Talley NJ Rath DM Zinsmeister AR Discriminant value of psychological distress, symptom profiles, and segmental colonic dysfunction in outpatients with severe idiopathic constipation Gut 1994 35 6 798 802 10.1136/gut.35.6.798 8020809 PMC1374882

[B36] Wong RK Palsson OS Turner MJ Levy RL Feld AD von Korff M Whitehead WE Inability of the Rome III criteria to distinguish functional constipation from constipation-subtype irritable bowel syndrome Am J Gastroenterol 2010 21 41 54 10.1038/ajg.2010.200 PMC3786710 20502449

[B37] Rao SS Tuteja AK Vellema T Kempf J Stessman M Dyssynergic defecation: demographics, symptoms, stool patterns, and quality of life J Clin Gastroenterol 2004 38 8 680 685 10.1097/01.mcg.0000135929.78074.8c 15319652

[B38] Rangnekar AS Morgan DR Knechtges P Saad RJ Fenner D Morris AM Chey WD Complaints Suggestive of Irritable Bowel Syndrome Are Common in Patients with Puborectalis Dyssynergia: An Under-Recognized Overlap Syndrome Gastroenterology 2008 134 A423

[B39] Camilleri M Inclusion criteria for pharmacodynamic and clinical trials in chronic idiopathic constipation: pitfalls in using Rome III for functional constipation Therap Adv Gastroenterol 2011 4 3 159 163 10.1177/1756283X11401773 21694799 PMC3105610

[B40] Glia A Lindberg G Nilsson LH Mihocsa L Akerlund JE Clinical value of symptom assessment in patients with constipation Dis Colon Rectum 1999 42 11 1401 1408 discussion 1408-1410 10.1007/BF02235036 10566527

[B41] Koch A Voderholzer WA Klauser AG Muller-Lissner S Symptoms in chronic constipation Dis Colon Rectum 1997 40 902 906 10.1007/BF02051196 9269805

[B42] Knowles CH Eccersley AJ Scott SM Walker S Reeves BD Lunniss PJ Linear Discriminant Analysis of Symptoms in Patients with Chronic Constipation: Validation of a New Scoring System (KESS) Dis Colon Rectum 2000 43 10 1419 1426 10.1007/BF02236639 11052520

[B43] McCrea GL Miaskowski C Stotts NA Macera L Varma MG Pathophysiology of constipation in the older adult World J Gastroenterol 2008 14 17 2631 2638 10.3748/wjg.14.2631 18461648 PMC2709058

